# Research on health information avoidance behavior and influencing factors of cancer patients—an empirical analysis based on structural equation modeling

**DOI:** 10.1186/s12889-024-21113-4

**Published:** 2024-12-31

**Authors:** Rui Zhu, Hui Zhao, Yun Yun, Yue Zhao, Weixian Wang, Lingmeng Wang, Wenjie Hou, Fuzhi Wang

**Affiliations:** 1 Bengbu Medical University, School of Nursing, Bengbu, China; 2 Bengbu Medical University, School of Health Management, Bengbu, China; 3 Bengbu Medical University, Longzi Lake Campus, Bengbu Medical University, No. 2600 Donghai Avenue, Bengshan District, Bengbu City, Anhui Province, China

**Keywords:** Cancer patients, Health information avoidance, Influencing factors, Structural equation modeling, Intermediary effect

## Abstract

**Objective:**

To explore the health information avoidance behaviors and influencing factors of cancer patients, and to construct a structural equation model to analyze the mediating roles of self-efficacy and negative emotions in the process of generating health information avoidance behaviors of cancer patients.

**Methods:**

A face-to-face electronic questionnaire was used to collect data. Applying a chi-square test and multivariate logistic regression model to analyze the role of different socio-demographic factors in influencing health information avoidance behavior of cancer patients; applying structural equation modeling to analyze the role mechanism of health information avoidance behavior of cancer patients.

**Results:**

The results of multivariate logistic regression analysis revealed that socio-demographic factors of per capita monthly household income, marital status, occupation, treatment modality, years of use of smart devices, and weekly hours of reading health information had an impact on health information avoidance behavior of cancer patients. All fit indices of the structural equation model were within acceptable limits,(CMIN/DF = 2.285,RMSEA = 0.045,CFI = 0.949,IFI = 0.949,RFI = 0.902,TLI = 0,942).The results of the mediating effect found that self-efficacy mediated the paths of information overload and privacy concern to health information avoidance behavior, respectively; negative emotions mediated the paths of information overload and privacy concern to health information avoidance behavior, respectively; and self-efficacy mediated the path from social support to health information avoidance behavior.

**Conclusion:**

Sociodemographic factors influencing cancer patients' health information avoidance behaviors include per capita monthly household income, occupation, treatment modality, number of years of smart device use, and number of hours per week reading health information. Self-efficacy and negative emotions mediated the analytic model of health information avoidance behavior in cancer patients, respectively.

## Background

The report published by the International Agency for Research on Cancer (IARC) of the World Health Organization shows that cancer is a specific chronic disease with high morbidity and mortality [1]. In 2022, there will be about 20 million new cancer cases globally, including about 4,824,700 new cases and 2,574,200 deaths in China [2]. The number of new cancer cases worldwide is expected to reach 22.2 million in 2030, with 5.81 million new cases in China.

The 53rd Statistical Report on the Development of the Internet in China shows that as of December 2023, the number of Internet users in China had reached 1.092 billion, with an Internet penetration rate of 77.5% [3]. With the development of Internet technology and electronic devices, most cancer patients seek health information through the Internet [4], which has become the main channel for cancer patients to obtain health information. However, when cancer patients are confronted with massive amounts of health information, issues such as information overload, information risk, and privacy leakage can trigger their health information search fatigue, which in turn causes negative emotions such as fear and anxiety, leading them to consciously avoid or delay accessing certain valuable health information, a phenomenon known as health information avoidance behavior [5].

Information avoidance first appeared in the field of psychology, where psychologist K. Seeny argued that information avoidance is any behavior in which people delay accessing or defending against information that may be needed in response to perceived stress or potential threat [6]. The results of a series of international studies in recent years have shown that factors affecting health information avoidance behavior in cancer patients include age, gender, health literacy, credibility of information, and negative emotions [7, 8]. In China, with the rapid development of AI technology and the widespread use of information mobile terminal devices by the public, health information avoidance behavior has become a factor that cannot be ignored in influencing public health [9]. For cancer patients, they often bear the stress of a disease that is difficult to cure and face a high risk of death. Both physical and psychological stresses make them more likely to develop health information avoidance behaviors in health information search [8]. However, research addressing the factors influencing health information avoidance behaviors of cancer patients in mainland China is lacking.

Health information avoidance, as an important class of behavior in behavioral psychology research, it is essential to select appropriate theoretical models to conduct relevant research. And the S–O-R theory provides methodological support for the development of such studies. The S–O-R theory, known as Stimulus-Organism-Response theory, was proposed by Mehrabian and Russell, the theory that the mechanism of human information activity is not a simple ‘stimulus–response’ model, in which there is an individual information internalization process, the individual receives external information stimuli, the information content to the individual psychological level, and produce cognitive responses, which in turn changes the individual information behavior [10]. In this theory, S denotes a stimulus, defined as some kind of object, event, or feature; O denotes internal response and is the mediating variable, the mechanism of mental transformation by which the organism internalizes external stimuli, which suggests that the organism's behavior is influenced by factors internal to it; R denotes the organism's relevant response behavior to the information content of the stimulus. A large number of previous studies have demonstrated the wide application of the S–O-R theory in explaining the influencing factors and mechanisms of public health management behaviors [11], consumer purchasing behaviors [12] and social behaviors [13]. Therefore, it is scientific and feasible to conduct research on public health information avoidance behavior based on the S–O-R theory.

In this study, we take cancer patients as the research object, apply quantitative research methods to carry out empirical research on cancer patients' health information avoidance behaviors and their influencing factors, explore the role of socio-demographic factors in influencing cancer patients' health information avoidance behaviors, and analyze the mediating effects of several factors in the process of health information avoidance behaviors based on the S–O-R model.

## Information and methodology

### Formulation of research hypotheses

This study is based on the S–O-R theoretical model to construct a research hypothesis on health information avoidance behavior of cancer patients.

**Fig. 1:**
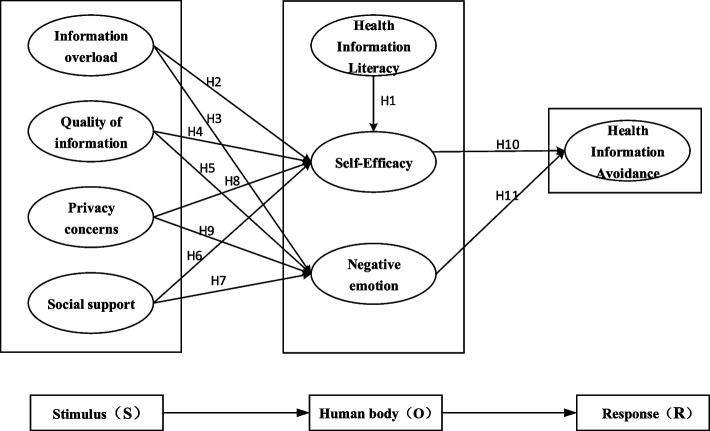
Analytical model of cancer patients' health information avoidance behavior in the age of smart media

The formulation of the research hypothesis included the following three steps: First, keywords such as health information avoidance behavior, health information literacy, and information quality were used to search the China Knowledge Network; Searching Health Information Avoidance Behavior、Negative Emotions Information Overload PubMed, Web of Science databases with subject terms such as. 122 health information avoidance studies were obtained (see Appendix 1 for literature inclusion, exclusion and search process criteria). Secondly, the abstracts of these literatures were carefully read to further identify the studies in the literature, and a total of 15 literatures were included in this study (see Appendix 2 for a list of literature characteristics). Third, research variables related to health information avoidance were extracted from the literature, and seven variables were selected for inclusion in the S–O-R model, resulting in 11 research hypotheses (see Table 1). Ultimately, an analytical model of cancer patients' health information avoidance behavior was constructed (see Fig. 1).

**Table 1:** Research hypotheses

Hypothetical	Research Hypothesis	Basis for assumptions
H1	Health information literacy is positively associated with self-efficacy in cancer patients	Heather Orom et al. [7]
H2	Information overload is negatively associated with self-efficacy in cancer patients	RAVINDRAN T et al. [14]
H3	Information Overload Positively Correlated with Negative Emotions in Cancer Patients	Song et al. [15]Haiyun Ma et al. [16] Yijing wang et al. [17] Saira Hanif Soroya [18]
H4	Information quality is positively associated with self-efficacy in cancer patients	Ye Chen et al.[19]Shuya Pan et al. [20] Elena Link [21]
H5	Information quality is negatively associated with negative emotions in cancer patients	Liuhan Zhan et al. [22]
H6	Social support is positively associated with self-efficacy in cancer patients	Amandeep Dhir et al. [23]
H7	Social support is negatively associated with negative emotions in cancer patients	Wen Gong et al. [24]
H8	Privacy concerns are negatively associated with self-efficacy in cancer patients	Amandeep Dhir et al. [23]
H9	Privacy Concerns Positively Correlated with Negative Emotions in Cancer Patients	Ye Chen et al. [19]
H10	Self-efficacy is negatively associated with health information avoidance behavior in cancer patients	Shuai Zhang et al. [25] Lihui Peng et al. [26]
H11	Negative emotions are positively associated with health information avoidance behaviors in cancer patients	Song et al. [27]Lihui Peng et al [28] Haiyun Ma et al. [16]

### Sample selection

This study was conducted on cancer patients in 1 tertiary hospital in Bengbu City, Anhui Province, using a two-stage sampling method. Stage 1: Simple random sampling was applied to obtain the research department in the oncology department of the First Affiliated Hospital of Bengbu Medical University. Stage 2: Eligible cancer patients were selected from the electronic medical records of the hospital information system using simple random sampling.

Sample inclusion criteria: (1) age 18 years or older; (2) histologically or pathologically confirmed diagnosis of cancer; (3) ability to independently use the internet/smart devices; and (4) informed and consent to participate in the study. Sample exclusion criteria: (1) patients with cognitive dysfunction or mental illness; (2) lack of basic literacy skills; and (3) withdrawal from the survey midway through.

### Survey instruments

In order to ensure the scientific validity of the study and the good reliability of the questionnaire, all the measurement items in this study were referred to existing, well-established scales and adapted to the context of this study. The survey covered eight areas: health information literacy, self-efficacy, negative emotions, information overload, information quality, privacy concerns, social support, and health information avoidance behaviors. All entries in the questionnaire were made on a Likert 5 scale, with a score of 1 to 5 indicating “strongly disagree (1)” to “strongly agree (5).” The survey instrument is shown in Appendix 3.

#### Health information literacy

The Everyday health information literacy (Everyday health information literacy) questionnaire, sinicized and revised by Luo Dan and other scholars, was used to evaluate the level of health information literacy of Chinese residents, including four dimensions and a total of 14 entries, with a Cronbach's alpha of 0.697 [29]. This study uses entries 1, 2 and 7 of these.

#### Self-efficacy

The General Self-Efficacy Scale (GSES), developed by Schwarzer et al., Perceptions or beliefs that were used to evaluate whether an individual can adopt adaptive behaviors in the face of challenges in the environment, with 10 entries and a Cronbach's alpha of 0.880 [30]. This study uses 1, 4, 5, 8, and 9 of these entries.

#### Negative emotion

The Health Information Avoidance (HIA) Scale for College Students, developed by scholars such as Shuai Zhang, has been used to evaluate the tendency of health information avoidance in the college student population, it includes 3 dimensions with 12 entries and a Cronbach's alpha of 0.951 [31]. This study used entries 1–4 of them.

#### Information overload

The Cancer Information Overload Scale (CIOS), developed by scholars such as Jensen, has been used to evaluate the extent to which individuals perceive cancer information overload, a total of 13 entries with a Cronbach's alpha of 0.770 [32]. This study uses entries 1–4 and 7 of them.

#### Quality of information

The HON Code of Conduct for medical and health Websites (HONcode evaluation scale), developed by the Swiss Health Online Foundation, is a tool used to evaluate the quality of online health information. The application of HONcode to evaluate the quality of online health information is based on eight principles, including eight guidelines such as authority, complementarity, confidentiality, and reasonableness [33].

#### Privacy concerns

Internet Information Privacy Concerns (IUIPC) questionnaire, developed by scholars such as Naresh K. Malhotra, was used to measure consumers' concern for information privacy and consisted of 3 dimensions with 10 entries, Cronbach's alpha为0.910 [34]. This study uses one of the collection dimensions.

#### Social support

Perceived social support scale (PSSS), developed by Zimet and other scholars, was used to evaluate the extent to which individuals feel supported by family, friends, and others, and consists of 3 dimensions with 12 entries, with a Cronbach's alpha of 0.930 [35]. This study uses 1, 3, 4, and 9 of these entries.

#### Health information avoidance

The Information Avoidance Scale (IAS), developed by Howell and other scholars, was used to evaluate the extent of individual information avoidance behaviors with 8 entries and a Cronbach's alpha of 0.890 [36]. This study uses 1, 2, 4, 6, and 8 of these entries. According to the rules for assigning scores on the scale, samples with a total score of 40% or less were considered to have no avoidance behavior, while the rest of the samples were considered to have avoidance behavior [37].

### Data acquisition

This study applied an electronic questionnaire to complete data collection by a person on a one-to-one basis. Prior to the start of the survey, all questionnaire entries and survey technical details were discussed in detail by the four investigators to ensure consistency in the research process. Accompanied by the medical and nursing staff of the department, a professionally trained investigator introduces the purpose and content of the survey to the patients, seeks their informed consent, and then invites them to participate in this survey in a quiet conference room. The survey was limited to 10–20 min per respondent. A gift ($8 value) was given to each respondent after all surveys were completed.

### Statistical methods

The chi-square test was used to analyze differences in cancer patients' willingness to engage in health information avoidance behaviors across sociological contexts; Multiple logistic regression models were used to analyze the role of different sociodemographic factors in influencing the health information avoidance behavior of cancer patients; SPSS software was applied to test normality of all continuous variables; Amos software was applied to construct structural equation modeling; and the Bootstrap method was applied for the mediation effect test. The initial organization of the data was done by applying Excel 2019 (Microsoft Inc. Washington, DC, USA), and data analysis was done using IBM SPSS Statistics (Ver. 26, IBM Inc. New York, USA). Data modeling was completed using Amos (Ver. 26).

## Results

### The current situation of health information avoidance behavior of cancer patients in the age of smart media

**Fig. 2:**
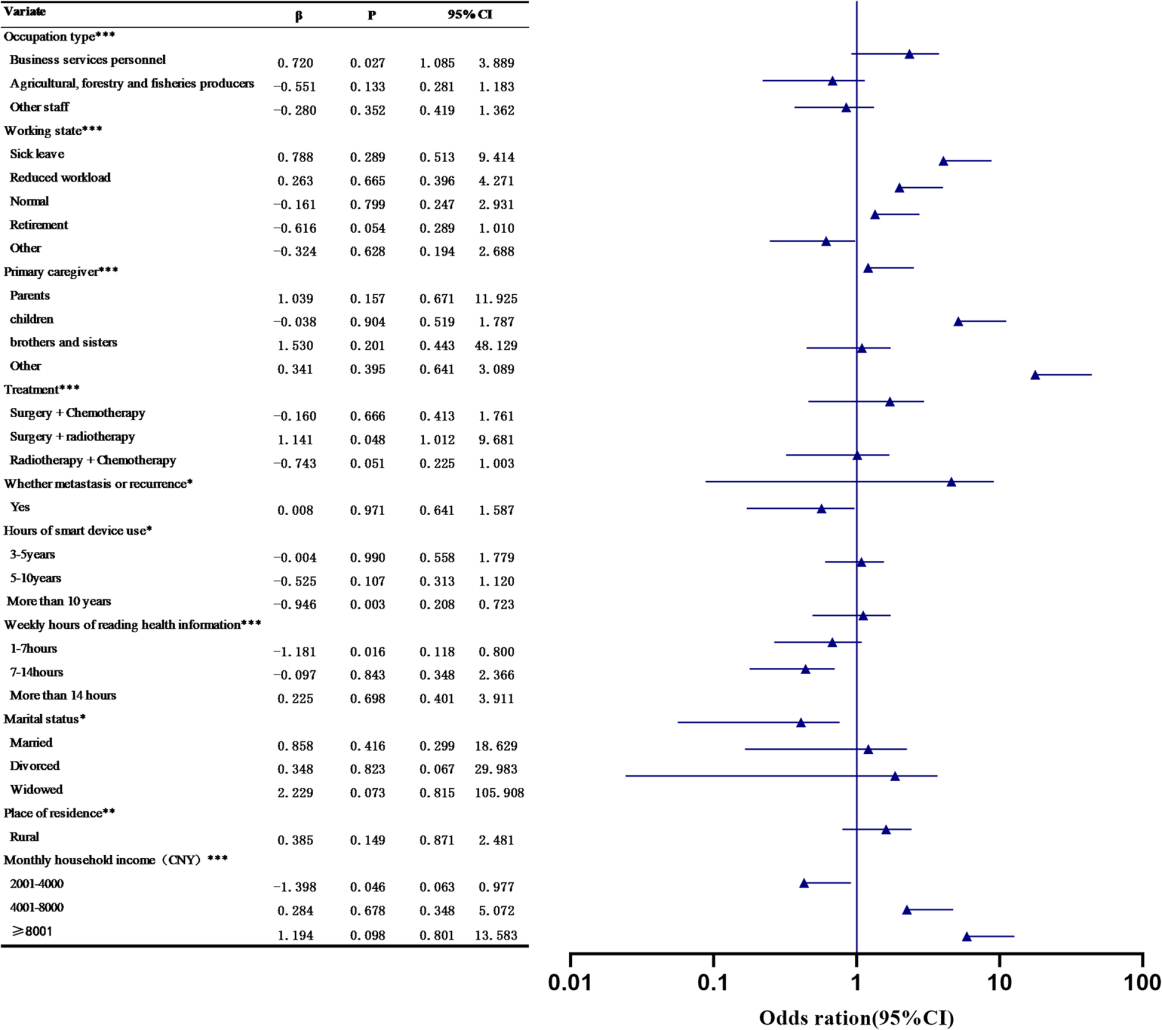
Results of chi-square test and regression-square analysis of health information avoidance behavior in cancer patients (*p < 0.05; **p < 0.01; ***p < 0.001)

The sociodemographic characteristics and medical consultations of the 643 respondents are shown in Table 2. Of the 643 respondents, 346 (53.8%) were cancer patients without health information avoidance behaviors, and 297 (46.2%) were cancer patients with health information avoidance behaviors. The results of the univariate analysis showed that there were significant differences in the health information avoidance behaviors of cancer patients across per capita monthly household income, marital status, work status, occupation, primary caregiver, treatment modality, whether metastasis or recurrence, number of years of use of smart devices, and the number of hours spent looking at the health information per week, as shown in Fig. 2.

**Table 2:** Basic characteristics of respondents

**Variate**		**n(%)**	**Variate**		**n(%)**
Gender	Male	288(44.8)		children	106 (16.5)
	Female	355(55.2)		brothers and sisters	71 (1.1)
Age group	20–40	46 (7.2)		other	45 (7)
	41–60	362 (56.3)	Treatment	surgeries	67 (10.4)
	≥61	235 (36.5)		Surgery + Chemotherapy	273 (42.5)
Marital status	Spinsterhood	6 (0.9)		Surgery + radiotherapy	54 (8.4)
	Married	610 (94.9)		Radiotherapy + Chemotherapy	249 (38.7)
	Divorced	4 (0.6)	Whether metastasis or recurrence	No	431 (67.0)
	Widowed	23 (3.6)		Yes	212 (33.0)
Place of residence	Urban	199 (30.9)	Presence of other chronic diseases	No	373 (58.0)
	Rural	444 (69.1)		Yes	270 (42.0)
Monthly household income(CNY)	≤2000	350 (54.4)	Religions	No	572 (89.0)
	2001–4000	182 (28.3)		Yes	71 (11.0)
	4001–8000	96 (14.9)	Hours of smart device use	Less than 3 years	191 (29.7)
	≥8001	15 (2.3)		3–5 years	151 (23.5)
Highest educational level	Primary	539 (83.8)		5-10 years	121 (18.8)
	Intermediate	71 (11.0)		More than 10 years	180 (28.0)
	High class	33 (5.1)	Weekly hours of reading health information	Less than 1 h	384 (59.7)
Occupation type	Professionals	139 (21.6)		1-7 h	161 (25.0)
	Agricultural, forestry and fisheries producers	159 (24.7)		7-14 h	61 (9.5)
	Business services personnel	35 (5.4)		More than 14 h	37 (5.8)
	Other staff	310 (48.2)	Type of medical insurance	Medical insurance for urban and rural residents	530 (82.4)
Type of Cancer	Tumors of the digestive system	241 (37.5)		Medical insurance for urban workers	112 (17.4)
	Tumors of the urinary system	201 (31.3)		Other	1 (0.2)
	Tumors of the respiratory system	148 (23.0)	Working state	not have	461 (71.7)
	Hematologic Tumors	21 (3.3)		sick leave	20 (3.1)
	Malignant tumors of the head and neck	15 (2.3)		Reduced workload	26 (4.0)
	Other	17 (2.6)		normal	20 (3.1)
Primary caregiver	spouses	469 (72.9)		retirement	98 (15.2)
	parents	16 (2.5)		Other	18 (2.8)

### Influential factors related to health information avoidance behavior of cancer patients in the smart media era

The results of the analysis of multiple logistic regression found that the sociodemographic factors of smart device usage of 10 years or more (β = -0.946), the length of reading health information of 1–7 h per week (β = -1.181), and the per capita monthly household income of 2001–4,000 yuan or more (β = -1.398) were found to be lower in the health information avoidance behaviors of cancer patients. In contrast, health information avoidance behavior was higher among cancer patients whose occupation was business or service worker (β = 0.720) and whose treatment was surgery and radiotherapy (β = 1.141), see Fig. 2.

### Reliability analysis

In this study, Cronbach's alpha coefficient and Combined Reliability (CR) were used to assess the indicators of reliability, and the values of Cronbach's alpha coefficient for each, variable are shown in Table 3. From the results in Table 3, it can be concluded that the Cronbach's alpha coefficient values as well as the CR values of the variables in this study are higher than 0.7, which indicates good reliability, and therefore the scale is considered to be highly reliable. The factor loadings and average extraction variance (AVE) for each variable are shown in Table 3. The AVE values for each variable were above 0.5, indicating that the measurement items of the questionnaire possessed good convergent validity.

**Table 3:** Reliability and validity analysis

Variate	Cronbach's Alpha	AVE	CR
Health Information Literacy	0.775	0.546	0.780
Quality of information	0.837	0.637	0.840
Privacy concerns	0.803	0.601	0.815
Social support	0.786	0.521	0.831
Information overload	0.887	0.570	0.888
Self-Efficacy	0.842	0.533	0.847
Negative emotion	0.900	0.754	0.910
Health Information Avoidance	0.939	0.754	0.939

In this study, the Pearson correlation coefficient and AVE square root value were chosen to determine the discriminant validity of the sample data. In general, when the AVE square root value in a dimension is significantly greater than the Pearson correlation coefficient between that dimension and the other dimensions, it indicates high discriminant validity. According to the results in the table, it can be seen that the AVE square root values of these eight dimensions are greater than the correlation coefficients between the dimension and the other dimensions, and it can be considered that the sample data has a very good discriminatory validity, as shown in Table 4.

**Table 4:** AVE square root values (Pearson correlation coefficients for each dimension)

	Health Information Avoidance	Negative Emotion	Self-Efficacy	Health Information Literacy	Social Support	Privacy Concerns	Quality of Information	Information Overload
Health Information Avoidance	0.868*							
Negative Emotion	0.224	0.868*						
Self-Efficacy	-0.187	-0.030	0.730*					
Health Information Literacy	-0.391	-0.043	0.507	0.740*				
Social Support	0.222	0.081	0.109	0.662	0.722*			
Privacy Concerns	-0.098	0.094	0.089	0.177	-0.141	0.775*		
Quality of Information	0.444	0.079	0.001	0.018	0.503	-0.285	0.798*	
Information Overload	0.066	0.142	-0.436	-0.155	-0.093	0.090	-0.044	0.755*

Note: * AVE square root value

### Model construction of influencing factors of cancer patients' health information avoidance behavior in smart media era

**Fig. 3:**
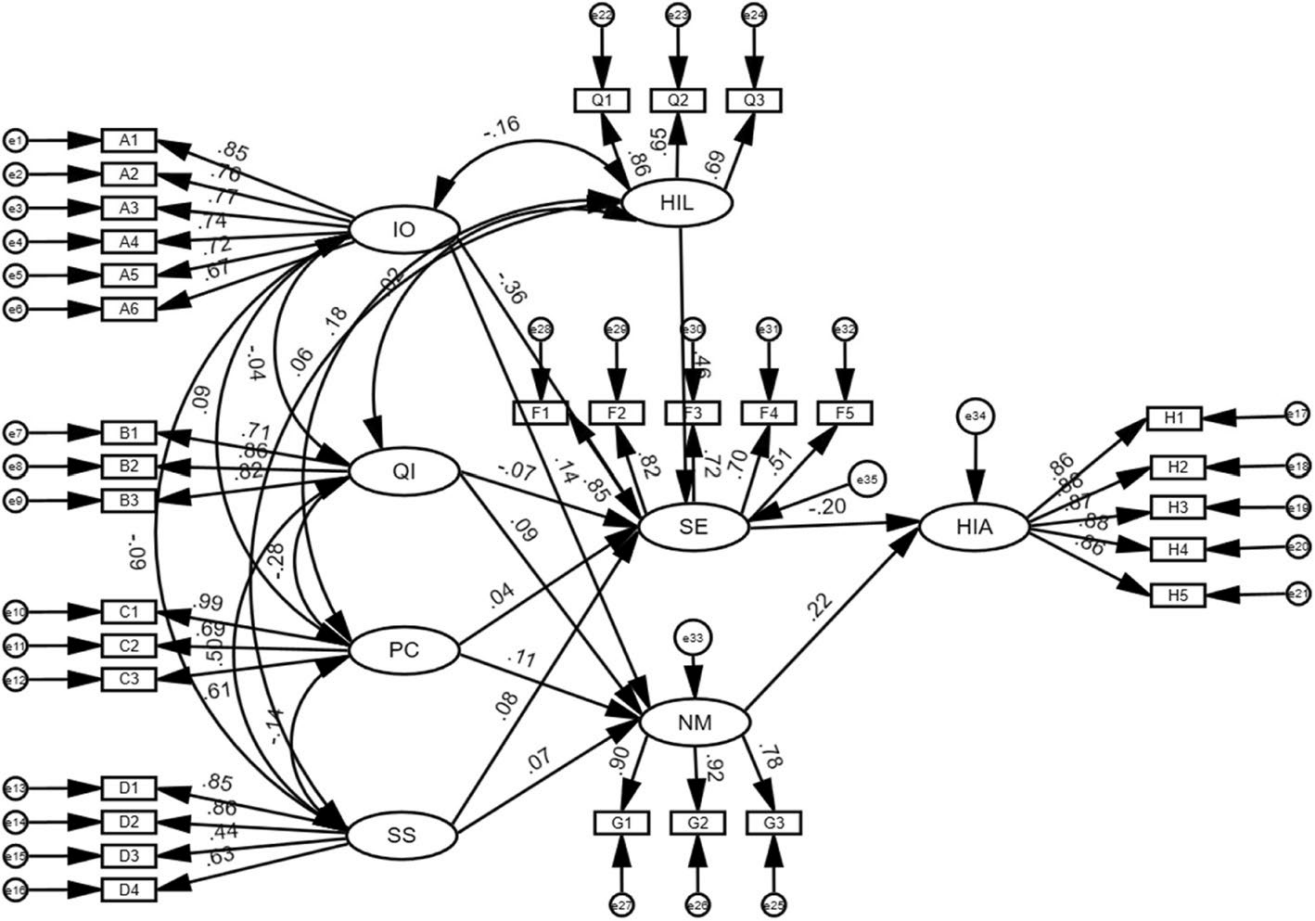
Model of factors influencing health information avoidance behavior of cancer patients

The model of factors influencing health information avoidance behavior of cancer patients is shown in Fig. 3.

### Model path test

The results of fitting the path analysis model for health information avoidance behavior are shown in Table 5. All the fit indices are within the acceptable range, indicating that the model is well fitted. The results of the path analysis of the model, are presented in Table 6. Health information literacy positively affects self-efficacy (β = 0.456, *p* < 0.001), and hypothesis H1 is valid. Information overload negatively affected self-efficacy (β = -0.361, *p* < 0.001), and hypothesis H2 was valid. Information overload positively affects negative affect (β = 0.140, *p* = 0.001), and hypothesis H3 holds; Privacy concerns positively influenced negative emotions (β = 0.114, *p* = 0.009), and hypothesis H9 was valid. Self-efficacy negatively affects health information avoidance behavior (β = -0.202, *P* < 0.001), hypothesis H10 is valid. Negative emotions positively influenced health information avoidance behavior (β = 0.220, *p* < 0.001), and hypothesis H11 was established. In contrast, hypotheses H4, H5, H6, H7, and H8 proposed in this study were not supported by the results of data analysis (*p* > 0.05).

**Table 5:** Model fit results

	CMIN/DF	RMSEA	CFI	IFI	RFI	TLI
Standard value	< 3.0	< 0.08	> 0.90	> 0.90	> 0.90	> 0.90
Actual value	2.285	0.045	0.949	0.949	0.902	0.942

**Table 6:** Results of path analysis

Hypothetical	Pathway	Standardized path factorβ	*P* Value	Results
H1	Health Information Literacy → Self-Efficacy	0.456	< 0.001	be tenable
H2	Information overload → Self-Efficacy	-0.361	< 0.001	be tenable
H3	Information overload → Social support	0.140	0.001	be tenable
H4	Quality of information → Self-Efficacy	-0.068	0.158	untenable
H5	Quality of information → Negative emotion	0.091	0.098	untenable
H6	Social support → Self-Efficacy	0.080	0.087	untenable
H7	Social support → Negative emotion	0.069	0.194	untenable
H8	Privacy concerns → Self-Efficacy	0.037	0.342	untenable
H9	Privacy concerns → Negative emotion	0.114	0.009	be tenable
H10	Self-Efficacy → Health Information Avoidance	-0.202	< 0.001	be tenable
H11	Negative emotion → Health Information Avoidance	0.220	< 0.001	be tenable

### Mediated effects test

In this study, the Bootstrap sampling method was chosen to conduct the mediation effect test to verify whether there is a mediation effect of negative emotion and self-efficacy between information overload, information quality, privacy concern, social support, and health information avoidance behavior, respectively, and the results are shown in Table 7.

**Table 7:** Results of the mediation effect test

Intermediary path	Effect size	LLCI	ULCI	P
Information overload → Self-Efficacy → Health Information Avoidance	0.066	0.032	0.107	< 0.001
Information overload → Negative emotion → Health Information Avoidance	0.291	0.167	0.420	< 0.001
Privacy concerns → Self-Efficacy → Health Information Avoidance	-0.031	-0.071	-0.008	0.008
Privacy concerns → Negative emotion → Health Information Avoidance	0.026	0.007	0.058	0.007
Social support → Self-Efficacy → Health Information Avoidance	-0.022	-0.052	-0.001	0.039

## Discussion

Sociodemographic factors affecting cancer patients include monthly household income per capita, occupation, treatment modality, years of smart device use, and number of hours per week reading health information. The results of the mediated effects analysis found that self-efficacy and negative emotions mediated the analytic model of health information avoidance behavior in cancer patients.

The results of the multivariate logistic regression analyses indicated that the number of hours per week spent reading health information and the number of years of smart device use play a key role in the health information avoidance behavior of cancer patients. Those cancer patients who moderately searched for health information on a weekly basis were less likely to engage in health information avoidance behaviors, whereas those who read health information for a longer period of time per week were conversely more likely to engage in health information avoidance behaviors. Possible reasons for this are that cancer patients tend to show more confidence and positively motivated health information perception and acceptance when they tend to be able to find the health information they need more easily, whereas cancer patients who search for health information for a long period of time tend to show deficits in their ability to locate and discriminate health information, and repeated querying experiences give them more anxiety, which gradually leads to health information avoidance behavior [38]. Another possible reason is that patients who have been using smart devices for a longer period of time tend to be more familiar with the ability to search for health information, and they tend to show more positive attitudes towards access to health information, resulting in a lower likelihood of health information avoidance behaviors. In addition, occupation and per capita monthly household income also showed a role in influencing the health information avoidance behavior of cancer patients. This is consistent with the findings of R. F. McCloud and other scholars [39]. This phenomenon may, on the one hand, be due to the high cost of antitumor treatment, the complexity of the treatment regimen, and the long treatment period [40], which may lead to those low-income families not being able to afford the high cost of treatment, and may even cause the patients to show negative attitudes towards cancer treatment [41], which may lead to their unwillingness to reach out to and inquire about health information. On the other hand, higher-paying occupations tend to mean that this group of patients is well educated and, in turn, exhibits higher levels of health information literacy. They also tend to show higher self-efficacy and lower negative emotions when faced with health problems, thus helping them to be more proactive in seeking and receiving health information. Finally, the way cancer patients are treated is also an important factor influencing health information avoidance behavior. Our findings suggest that health information avoidance behavior is higher among cancer patients whose treatment modality is surgery and radiotherapy. This may be due to the fact that death is the most sensitive topic in the context of traditional Chinese culture. Coupled with the influence of traditional filial piety, people try their best to save their loved ones when they are suffering from incurable diseases, even if the results are minimal [42]. However, since cancer is generally difficult to cure, cumbersome treatments and high financial outlays result in both physical and psychological damage to the patient, which in turn can further lead to health information avoidance behaviors.

The results of the path analysis of the factors influencing health information avoidance behavior in cancer patients showed that health information literacy had a positive effect on self-efficacy in cancer patients. The possible reason for this is that patients with higher health information literacy tend to have a clearer understanding of the health information they need and know how to accurately access, rationally use, and screen health information, and the more frequent their online health information searching behaviors will be [43]. Second, our findings found that information overload negatively affects both negative emotions and self-efficacy in cancer patients. It's understandable. With the rapid development of information technology, the amount of health information carried on the Internet has been increasing. On the one hand, the excessive amount of health information can cause patients' cognitive abilities to decline, which can lead to patient boredom [44]. On the other hand, cancer patients often find it difficult to cope with the huge amount of health information available on the Internet, which may lead them to question their ability to process health information, undermine their motivation and self-confidence in searching for health information, and consequently lead to wrong health decisions by the patients. Furthermore, our findings suggest that privacy concerns are positively associated with negative emotions in cancer patients. This may be due to the fact that patients are often asked to fill in sensitive personal information when using smart devices to search for health information, but patients lack trust in third-party policies to protect the security and privacy of their personal information, which can lead to negative emotions such as anxiety and worry [45]. Finally, our results found that cancer patients with high self-efficacy were less likely to develop health information avoidance behaviors, whereas patients with high negative affect were more likely to develop health information avoidance behaviors. This suggests that patients' higher self-efficacy allowed them to show more confidence in searching for health information and willingness to participate in disease management, which in turn further contributed to their receptivity to health information. In contrast, cancer patients who are bored with health information tend to be negative about accessing health information [46], which increases the likelihood that they will develop health information avoidance behaviors.

In our study, the quality of information did not show an influential role on self-efficacy and negative emotions in cancer patients, which may be related to the age of the sample and the study population. The respondents of this study were mainly middle-aged and elderly, for whom distinguishing the authenticity of health information on the Internet is a very difficult task, implying that they may not be too concerned about the quality of health information [47]. In addition, social support did not reflect the role of influencing self-efficacy and negative emotions of cancer patients. This may be due to the fact that cancer patients require long-term treatment, and the high medical expenses have forced their family members to take on more work tasks in order to alleviate the financial pressure on the family [48]. Reduced family presence makes it difficult for them to get help and support from their family members in terms of health information referrals.

The results of the mediating effect of health information avoidance behavior in cancer patients suggest that negative emotions in cancer patients mediate the relationship between information overload, privacy concerns, and health information avoidance behavior. Self-efficacy in cancer patients mediates information overload, privacy concerns, social support, and health information avoidance behaviors. In conjunction with the S–O-R theory, an individual, when stimulated in the external environment, will first change his or her internal state before further influencing the occurrence of behavior [10]. In the process of patients' health information acquisition and utilization, self-efficacy and negative emotions, as a comprehensive manifestation of patients' internal cognitive and emotional changes, reflect the degree of patients' confidence in their own health information-processing ability, and play a mediating role between various external stimuli faced by patients and health information avoidance behaviors. With the rapid development of digital technology, the huge amount of information on the Internet not only makes it difficult for patients to search for the health information they need, but also makes patients who are unaware of third-party privacy protection policies worry about the leakage of their sensitive personal information, and these reasons can lead to the generation of negative emotions and the reduction of self-efficacy of the patients, which will increase the possibility of the occurrence of health information avoidance behaviors [49]. For cancer patients who have difficulty using smart devices to search for health information, the lack of timely social support can cause a decrease in self-efficacy, which in turn promotes health information avoidance behaviors [50].

## Research values

The results of this study provide new perspectives for understanding the mechanisms underlying the occurrence of health information avoidance behaviors in cancer patients and provide important research ideas for developing targeted interventions in the future.

To reduce the incidence of health information avoidance behaviors among cancer patients, we offer the following recommendations: First, medical staff should regularly conduct training activities to enhance the health information application capacity of cancer patients, introducing reliable health knowledge science platforms and ways to use smart devices to search for health information, in order to improve their health information literacy and self-efficacy. Second, optimizing health information searching methods and popularizing privacy protection policies are also meaningful in reducing the occurrence of health information avoidance behaviors among cancer patients. Through education and training to introduce patients to accurate ways of searching for health information and the privacy protection features of the platforms, so that patients can accurately search for the health information they need and clearly understand that their sensitive personal information is protected by the third-party platforms, which can help to minimize the negative emotions caused by information overload and privacy concerns during the process of searching for health information by cancer patients. Finally, it is necessary to provide social support for cancer patients, and the role of the younger generation of “digital natives” as the promoters of health information literacy in the family should be fully utilized, and children should be encouraged to take the initiative to communicate and share their health knowledge with their elders, and to assist in the use of electronic devices, so as to enhance their self-efficacy.

## Conclusion

Sociodemographic factors influencing health information avoidance behaviors among cancer patients include monthly household income per capita, occupation, treatment modality, years of smart device use, and number of hours per week reading health information. The results of the analysis of the mediation effect showed that: Self-efficacy mediated the pathway from information overload to health information avoidance behavior (β = 0.066, *p* < 0.001); Self-efficacy privacy concerns mediated the pathway to health information avoidance behavior (β = -0.031, *p* = 0.008); Negative emotions mediated the pathway from information overload to health information avoidance behavior (β = 0.291, *p* < 0.001); Negative emotions mediated the path from privacy concerns to health information avoidance behaviors (β = 0.026, *p* = 0.007); Self-efficacy mediated the pathway from social support to health information avoidance behavior (β = -0.022, *p* = 0.039). Future studies should conduct multicenter cross-sectional investigations of health information avoidance behaviors of cancer patients, pay more attention to health information avoidance behaviors of cancer patients, and conduct relevant intervention studies.

## Limitations

There are some limitations to this study. First, China is a multiethnic country, but limited in energy and time, the sample in this study was from only one city and mostly middle-aged and elderly cancer patients, lacking analysis of the all-age and ethnic minority populations. Second, the factors influencing information avoidance behavior are diverse and complex; the role of seven factors influencing the health information avoidance behavior of cancer patients was discussed in this study, and more factors can continue to be explored in the future to influence the health information avoidance behavior of cancer patients. Third, this study was cross-sectional and could not answer the question of the developmental trends of the many influences acting on the change in health information avoidance behavior of cancer patients. Fourth, this study used a face-to-face electronic questionnaire to collect data, which may have led to recall bias in patients' responses. In the future, the acquisition of research data can be diversified, and emerging experimental methods such as eye movements and emotions can be introduced to improve the objectivity of the collected data. Despite these limitations, the relevant findings of this study still have a positive effect on reducing the incidence of health information avoidance behaviors in cancer patients and helping them to obtain more valuable health information.

## Data Availability

No datasets were generated or analysed during the current study.

## Appendix 1

Literature inclusion, exclusion and search process criteria

## Appendix 2

A list of literature characteristics

## Appendix 3

The survey instrument
